# Supplementary material for: The impact of the COVID-19 pandemic on mental health in the general population: a comparison between Germany and the UK
Source: BMC Psychol. 2021 Apr 24;9:60. doi: 10.1186/s40359-021-00565-y (PMC8064888; doi:10.1186/s40359-021-00565-y)
Supplement: Supplementary file 1 — Additional file 1. Supplementary materials showing additional figures illustrating differences between countries described in Table 1 (demographics and COVID-19 exposure and impact) as well as differences in clinical score (GSI, SPQ, and SPQ subdimensions). [file 40359_2021_565_MOESM1_ESM.pdf]

Supplementary materials

The impact of the COVID-19 pandemic on mental health in the general population – a comparison between Germany and the UK

Franziska Knolle[1,2], Lisa Ronan[2], Graham K. Murray[2,3]

[1] Department of Neuroradiology, Klinikum rechts der Isar, TUM, München, Germany

[2] Department of Psychiatry, University of Cambridge, Cambridge UK

[3] Cambridgeshire and Peterborough NHS Foundation Trust, Cambridge, United Kingdom

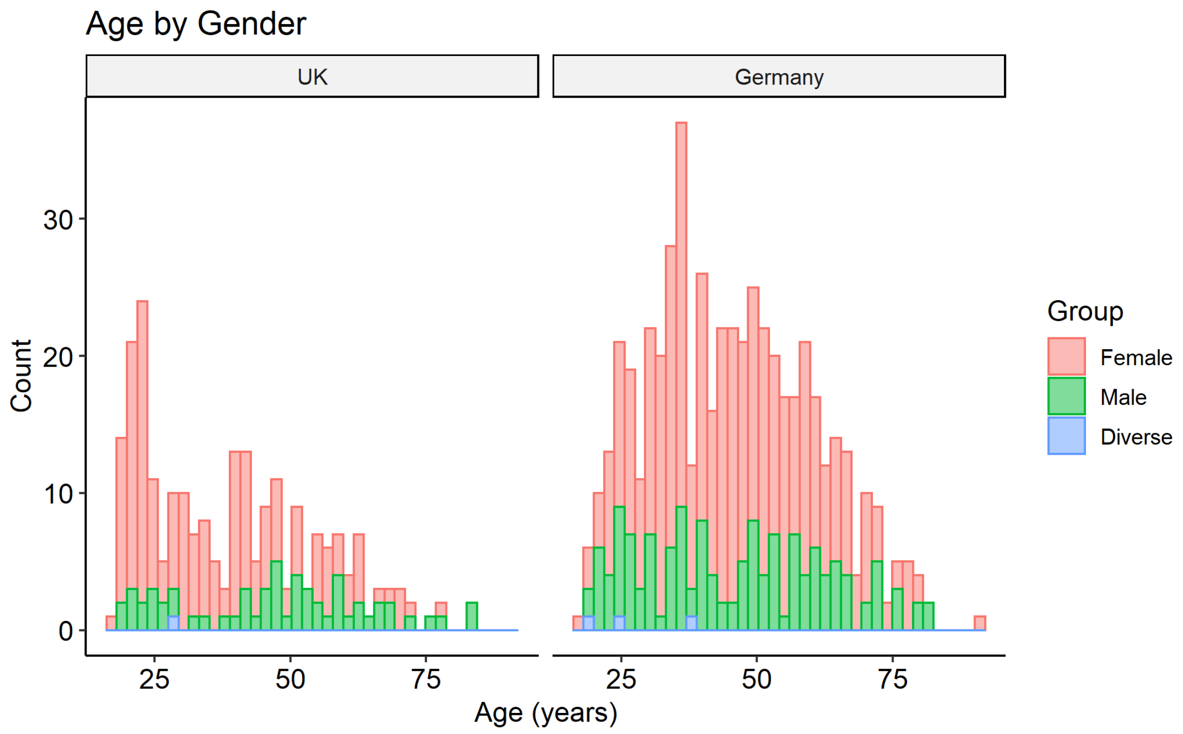

Suppl. Figure 1. Distribution of age by gender for the UK and Germany separately.

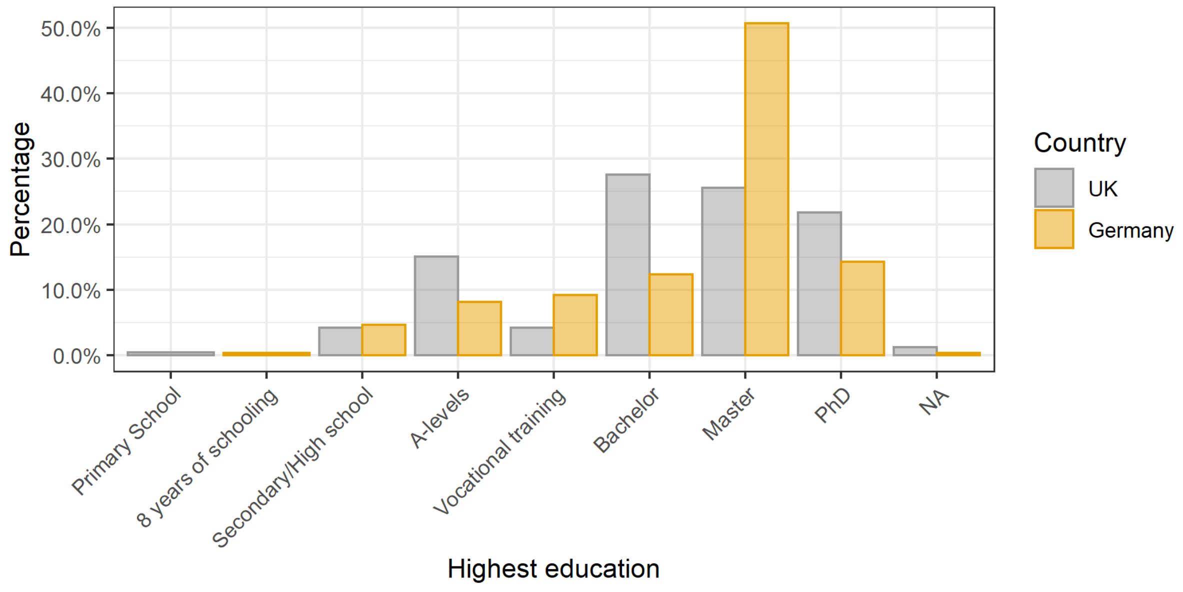

Supp. Figure 2. Distribution of highest personal education by country.

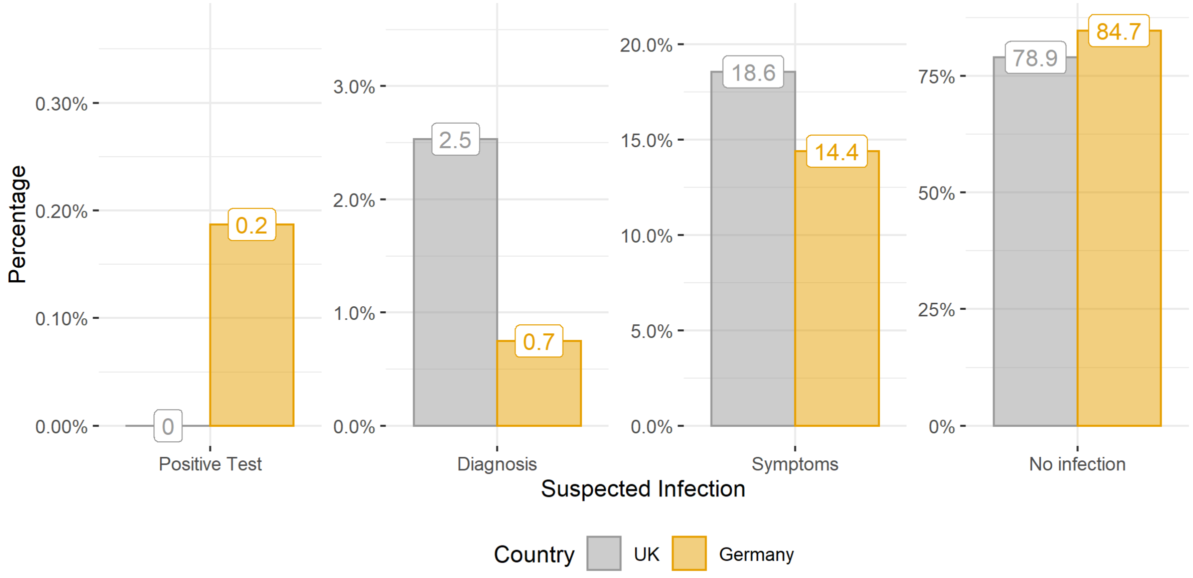

Supp. Figure 3. Percentage of suspected infection by country, presented in varying scales.

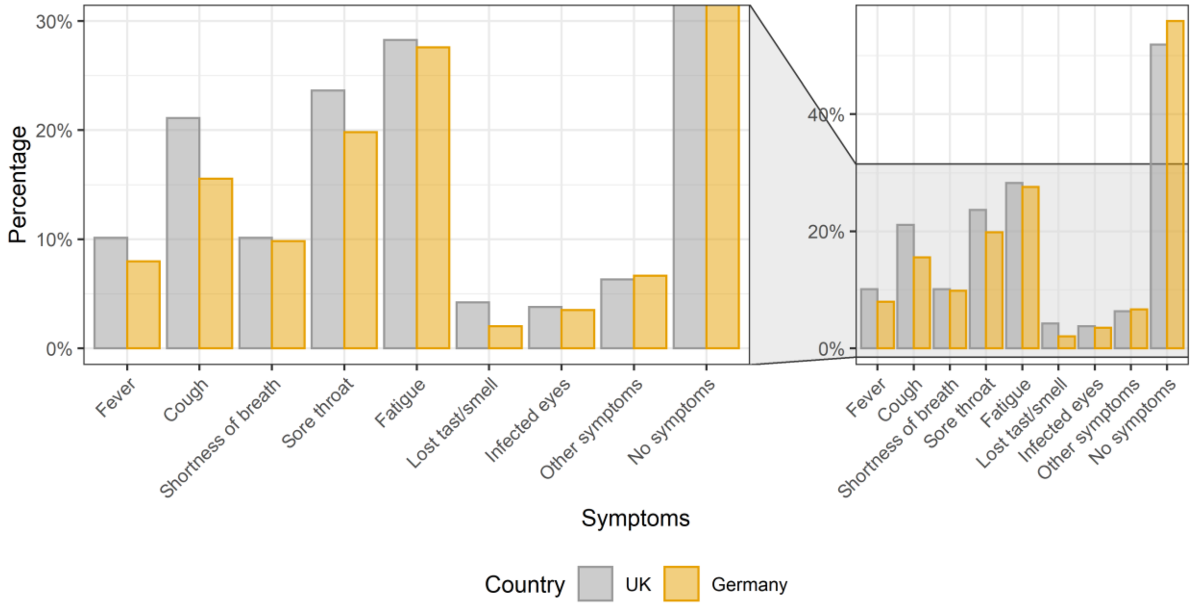

Supp. Figure 4. Distribution of symptoms by country, with close-up presented in the left panel.

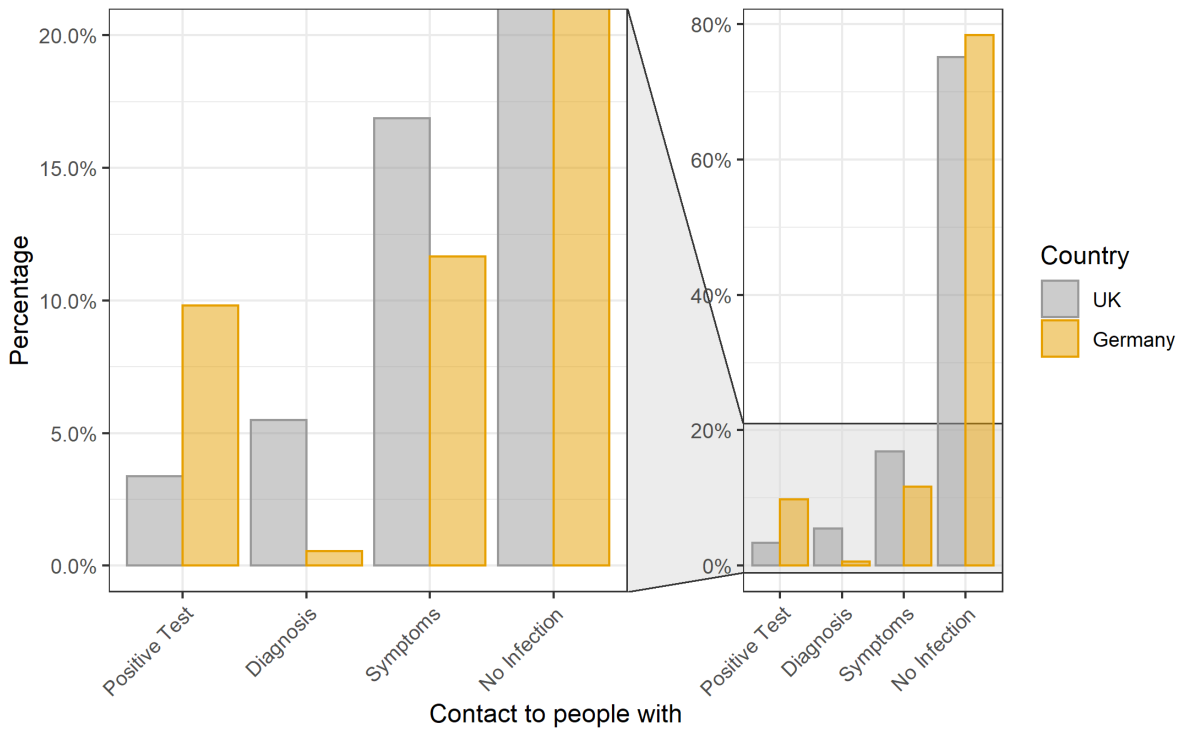

Supp. Figure 5. Distribution of contacts to people with possible infections by country, with close-up presented in the left panel.

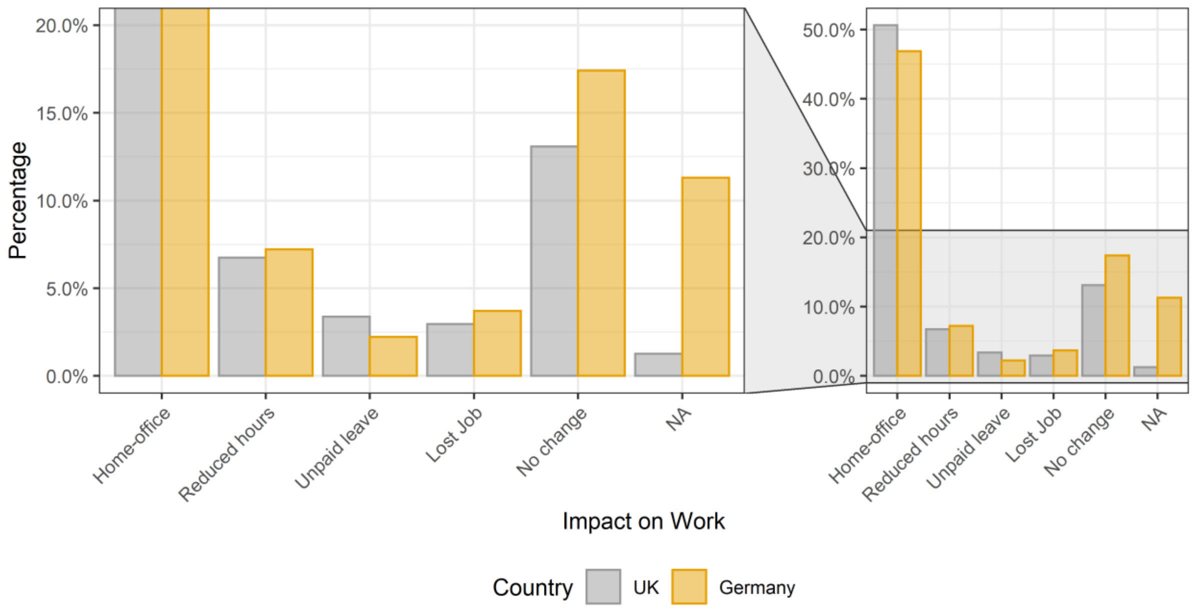

Supp. Figure 6. Impact on working situation by country, with close-up presented in the left panel.

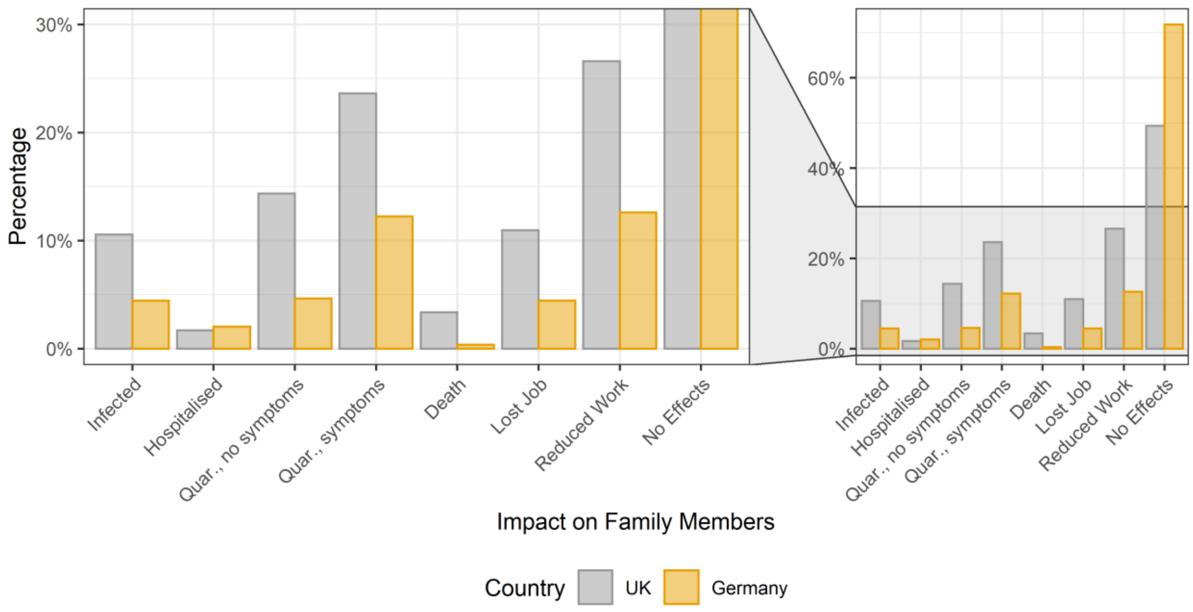

Supp. Figure 7. Impact on family by country, with close-up presented in the left panel.

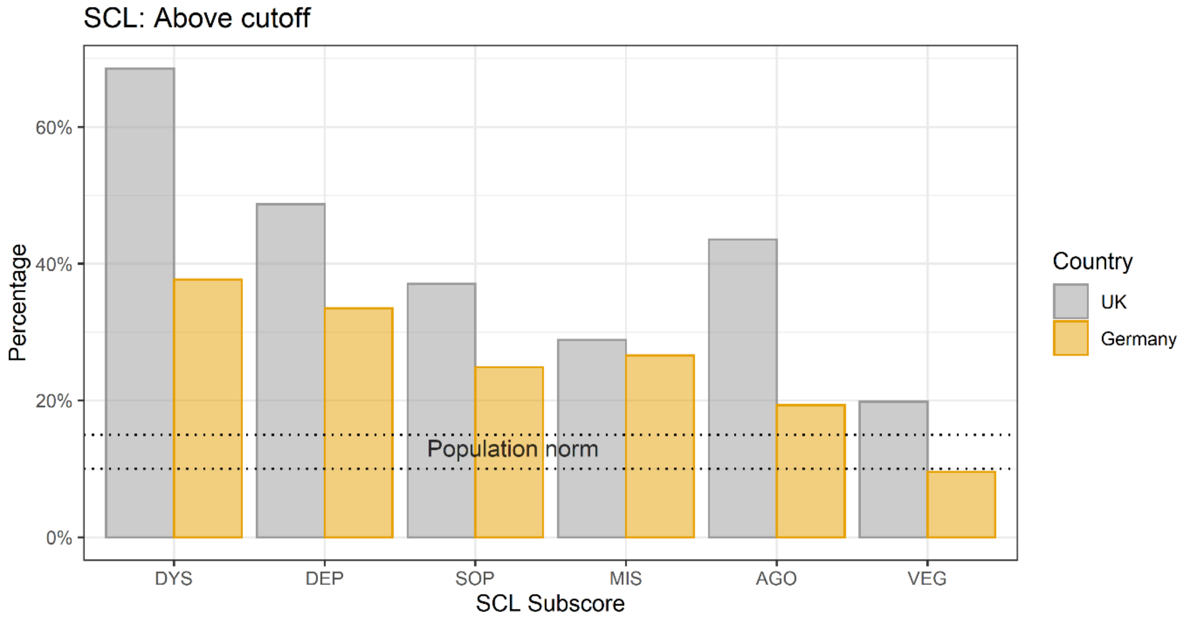

Supp. Figure 8. Percentage of responders above clinical cut-off separately for UK and Germany. Dotted lines represent the percentage of the norm population above threshold. DYS: dysthymic symptoms, DEP: depressive symptoms, SOP: symptoms of social phobia, MIS: symptoms of mistrust, AGO: agoraphobic symptoms, VEG: vegetative symptoms.

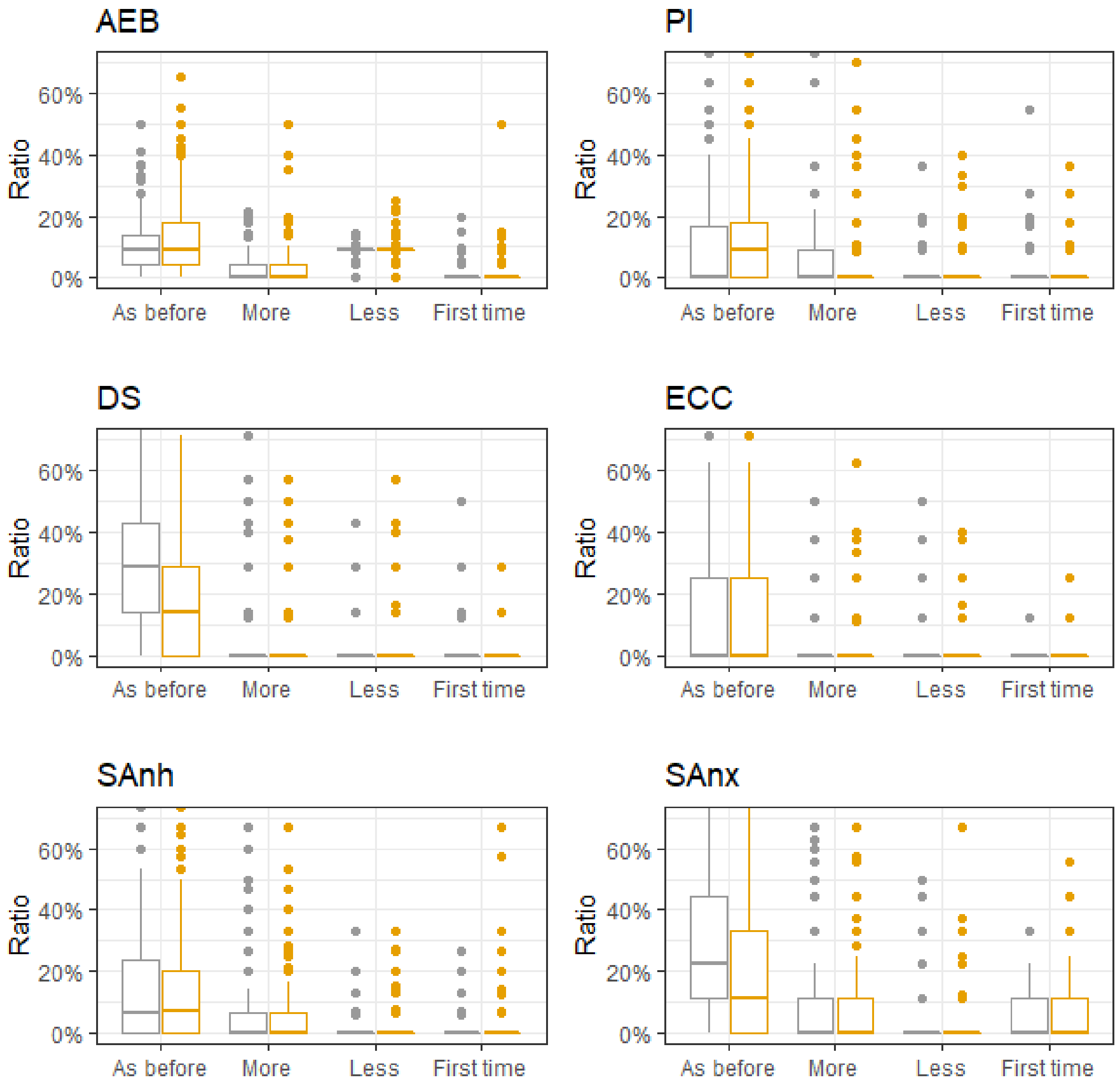

Supp. Figure 9. Each boxplot shows the subjective change of one of the sub-dimensions (experiences and beliefs (AEB), social Anhedonia (SAnh), paranoid ideation (PI), social anxiety (SAnx), eccentricity (Ecc), and disorganised speech (DS)) of the SPQ during the pandemic, separately for Germany (yellow-orange) and the UK (grey).

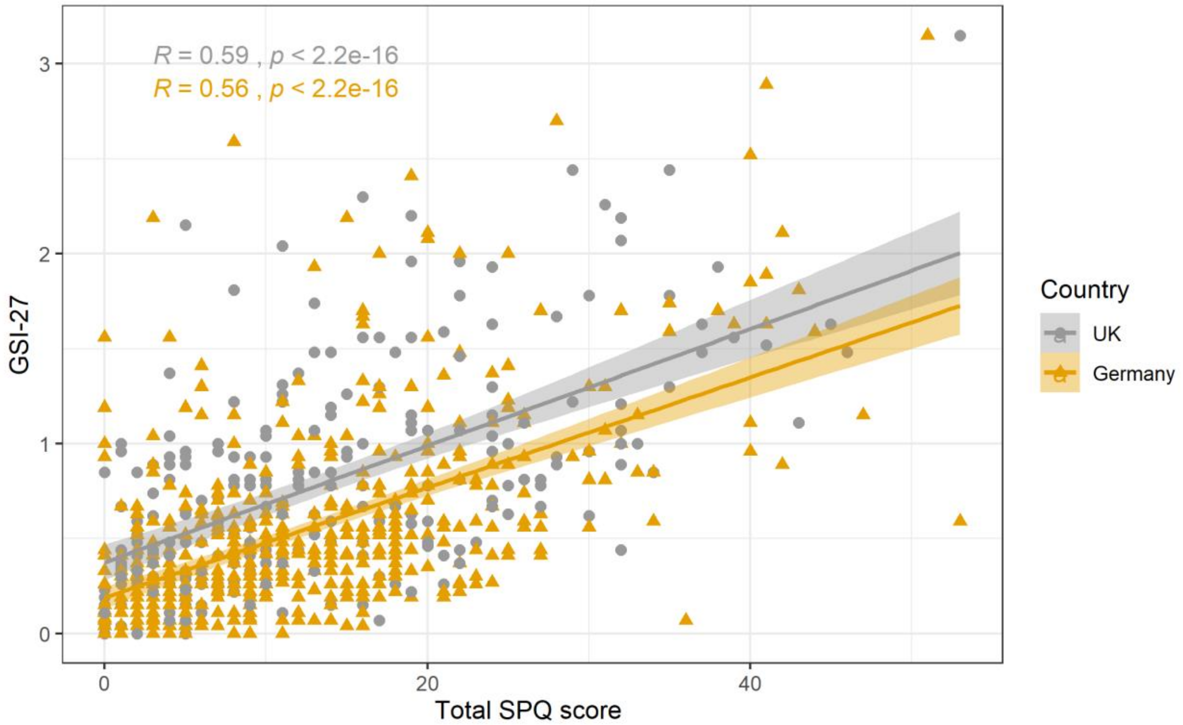

Supp. Figure 10. Pearson correlation between GSI-27 and total SPQ score, separately per country. GSI-27 and SPQ total reveal a strong positive correlation.
